# A community pharmacist medicines optimisation service for patients with advanced cancer pain: a proof of concept study

**DOI:** 10.1007/s11096-019-00820-8

**Published:** 2019-04-09

**Authors:** Zoe Edwards, Michael I. Bennett, Alison Blenkinsopp

**Affiliations:** 1 School of Pharmacy, Faculty of Life Sciences, University of Bradford, Richmond Building, Richmond Road, Bradford, West Yorkshire BD7 1DP UK; 2 University of Leeds, Leeds, UK

**Keywords:** Cancer, Community pharmacy, Medicines optimisation, Pain, Palliative care, Remote consultation, United Kingdom

## Abstract

*Background* Patients with advanced cancer commonly experience pain and it is least controlled in community settings. Community pharmacists in the UK already offer medicines optimisation consultations although not for this patient group. *Objective* To determine whether medicines consultations for patients with advanced cancer pain are feasible and acceptable. *Setting* Community-dwelling patients with advanced cancer pain were recruited from primary, secondary and tertiary care using purposive sampling in one UK city. *Methods* One face-to-face or two telephone delivered medicines optimisation consultations by pharmacists were tested. These were based on services currently delivered in UK community pharmacies. Feedback was obtained from patients and healthcare professionals involved to assess feasibility and acceptability. *Main outcome measure* Recruitment, acceptability and drug related problems. *Results* Twenty-three patients, (range 33–88 years) were recruited, 19 completed consultation(s) of whom 17 were receiving palliative care services. Five received face-to-face consultations and 14 by telephone during which 47 drug related problems were identified from 33 consultations (mean 2.5). Advice was provided for 34 drug related problems in 17 patients and referral to other healthcare professionals for 13 in 8 patients, 2 patients had none. Eleven patients returned questionnaires of which 8 (73%) would recommend the consultations to others. *Conclusion* The consultations were feasible as patients were recruited, retained, consultations delivered, and data collected. Patients found the 20–30 min intervention acceptable, found a self-perceived increase in medicines knowledge and most would recommend it to others. Community pharmacists were willing to carry out these services however they had confidence issues in accessing working knowledge. Most drug related problems were resolved by the pharmacists and even among patients receiving palliative care services there were still issues concerning analgesic management. Pharmacist-conducted medicines consultations demonstrate potential which now needs to be evaluated within a larger study in the future.

## Impacts on practice

- Pharmacist-delivered medicines consultations are feasible and acceptable to cancer patients and have the potential to benefit clinical care.
- Even for patients under specialist palliative care services, unmet medicines-related needs can be identified by pharmacists.
- Access to pharmacist care for patients with advanced cancer who are not able to visit the pharmacy in person should be improved.

## Introduction

Over half of patients with advanced cancer will experience poorly controlled pain during their last year of life [1, 2]. Only 18% of patients at the end-of-life living in the community describe their pain as well controlled compared with 38% of patients in hospitals and 63% of patients in hospices [3]. Often, community-based patients feel they lack support with their medicines taking and accept experiencing pain [4].

Community Pharmacists are the healthcare professional seen most frequently by patients with cancer (along with community nurses) [5] and are often available in every locality without an appointment. Community pharmacies may be in or near family doctor practices or sometimes in shopping centres or supermarkets and could potentially be an accessible source of medicines support for patients with cancer pain. Medicines optimisation services are provided by community pharmacists in the UK, USA, Australia and New Zealand with the aim of helping patients get the most benefit possible from the medicines they have been prescribed [6–10]. In the UK the two most common are detailed in Table 1.

**Table 1:** Medicines optimisation services provided by community pharmacies in England and Wales

Service	Medicine use review (MUR)	New medicine service (NMS)
Which patients?	70% must be targeted towards specified patient disease groups (not including cancer). The remainder may be carried out with any other patient [9]. They must be carried out in the patients usual community pharmacy [9]	Patients prescribed new medicines for specified long-term conditions [10]
Method of delivery	One consultation, usually face-to-face but telephone possible with relevant permissions [9]	Patient can choose face to face or telephone delivery. Up to three consultations: Initial advice, intervention and follow-up consultations usually by telephone [10]
Use in patients with cancer	Research indicates rarely provided [11]	Cancer not one of the specified long-term conditions [10]
Reimbursement	£28 [9]	Between £20 and £28 depending on number provided [10]

The World Health Organization Pain ladder provides stepwise guidance for adult pain management [11]. Severe pain can usually be controlled by regular dosing of simple pain killers, adjuncts and sustained-release pain medication with top-up or breakthrough doses in-between but patients need to understand their medicines to gain optimum benefit from them [12, 13]. Pharmacist medicines consultations have been shown to increase patient knowledge and have associated improvements in medicines adherence [14, 15].

Although community pharmacist medicines consultations are rarely carried out with patients experiencing cancer pain, several studies have investigated the contributions they could make [16–19]. Outcome measures included the quantity of Drug Related Problems (DRPs), recommendations made and an assessment of the appropriateness of recommendations [16–20]. A DRP can be defined as an event or circumstance involving drug therapy that actually or potentially interferes with desired health outcomes [21]. Patient’s perspectives are often difficult to obtain post-intervention from patients with advanced disease due to rapid deterioration and only one study included this [18, 22]. A recent systematic review showed that pharmacist educational interventions are potentially beneficial for patients with cancer pain but further research is needed in this area [23].

Lack of pharmacist confidence to provide services for patients with cancer has been identified as a barrier to service provision [24–26]. All previous studies either employed a specialist palliative care pharmacist or provided some sort of additional training, although the content and evidence-base of training was not always reported [16–19].

## Aim

To evaluate pharmacist medicines consultations for patients with advanced cancer and to ascertain their feasibility and acceptability.

### Ethics approval

Ethical permission was granted from Leeds West National Health Service and Bradford University Ethical Committee in October 2014 (14-YH-1126 141015). This study was part of the larger Improving the Management of Pain from Advanced Cancer in the Community (IMPACCT) study.

## Method

### Recruitment site identification

Research ready family doctor practices with practice pharmacists^1^ were identified and approached. Recruitment commenced in November 2015 and continued until March 2017.

The recruitment process was iterative responding to the levels of identification and recruitment of patients. New and refined recruitment methods were developed, and the local hospital and hospice were invited to be recruitment sites towards the end of the study.

### Community pharmacist recruitment

The 10 pharmacies closest to the practices recruited were identified (four from national multiples and six independents) and contact was made with owners or head offices. Nine agreed to take part with one independent stating lack of interest. An interactive briefing session for the pharmacists was developed based on the specific needs of community pharmacists found in previous studies [24–26]. Information from a specialist nurse and a cancer support charity was delivered and role plays were carried out with a cancer survivor from our Patient and Public Involvement (PPI) group. Pharmacists and support staff representing all community pharmacies attended the training which was well received.

Patients who used one of the study pharmacies were offered either a telephone-based Medicine Use Review (MUR) or a face-to-face MUR (see Table 1).

Patients who did not use one of the study pharmacies were offered telephone consultations from the Research Pharmacist (RP) using the New Medicine Service (NMS) format allowing the same issues to be discussed over a series of two telephone consultations (see Table 1). Our earlier research showed that patients with advanced cancer were willing to try this [4].

### Patient recruitment

In family doctor practices, searches using the study inclusion criteria (see Box 1) were conducted in electronic systems by practice pharmacists. Community pharmacists, district nurses and family doctors were asked to refer suitable patients to the GP practice, where the practice pharmacist screened them and confirmed suitability with their GP. Oncology research nurses at the local hospital were asked to conduct outpatient clinical records searches. The hospital outpatient pharmacy was asked to refer patients to the research nurses. Patients from primary and secondary care recruitment were then invited to take part by letter.

**Box 1:** Recruitment criteria

1. Aged over 16 years old 2. Have advanced cancer^a^, are aware of their diagnosis and are suffering from pain 3. Been given a prescription for opioids^b^ 4. Have not been prescribed anticipatory medicines^c^ and are therefore not in the last days of life 5. Have the capacity to provide informed consent and complete questionnaires before and after the consultation	

^a^Patients with advanced cancer are defined as those with metastatic cancer with histological, cytological or radiological evidence AND/OR those receiving anti-cancer therapy with palliative intent

^b^Opioids are codeine, codeine and paracetamol, codeine and ibuprofen, dihydrocodeine, paracetamol and hydrocodeine, tramadol, tapentadol, morphine, fentanyl, buprenorphine, diamorphine, hydromorphone, methadone and oxycodone

^c^Anticipatory medicines are medicines which are often used to control symptoms in the last days of life. These are usually prescribed in a package as this time approaches

The hospice research nurse, together with the study RP undertook recruitment in the local hospice. Patients about to be discharged or attending outpatient clinics were introduced to the study by their nurse. Those who expressed interest were provided with written information and consented face-to-face by the RP.

All patients gave written consent.

### Sample

As this was a proof-of-concept study a formal sample size was not required but we aimed to recruit 25 patients as this was adequate to assess whether it was possible to recruit and retain patients, whether the consultations were deliverable and acceptable to patients and healthcare professionals involved.

### Medicines optimisation consultations

The length of each consultation was noted by the pharmacist and any recommendations made to the patient and/or the prescriber were documented contemporaneously, in line with usual practice for NMS and MUR. Consultation records used a study code and did not contain any patient identifiable information.

### Patient and pharmacist feedback

Baseline and post-consultation follow-up patient questionnaires were developed based on validated pain measurement tools used in the IMPACCT study [27, 28]. Drafts were piloted with the study PPI group and feedback was obtained. The questionnaire included quantitative and qualitative questions about levels of pain (worst experienced in the last 24 h and at time of questionnaire completion on a 0–10 scale (0 = no pain to 10 = pain as bad as they could imagine). Self-reported knowledge information was also collected. The baseline questionnaire was posted before the consultation with a stamped addressed return envelope. The follow-up questionnaire was sent 2 weeks after the final consultation and included additional questions on self-perceived benefit from the consultation and whether the patient would recommend the medicines consultation to others. Questionnaires are available on request from the author.

### Data analysis

Medicines consultation records were coded by the RP using the Pharmaceutical Care Network Europe (PCNE) classification which is a validated system developed by experts to document DRPs, their cause and action taken following their identification [21].

Information from the baseline and follow-up questionnaires was collated and analysed using Excel spreadsheets.

### Feasibility

This will be assessed by whether it was possible to recruit and retain patients throughout the study and whether it was possible to train community pharmacists to deliver consultations.

### Acceptability

A theoretical framework which was developed based on systematic reviews involving acceptability and inductive and deductive reasoning of reviews and literature is available [29]. The framework details acceptability before (prospective), during (concurrent) and after the intervention (retrospective). This was adapted to assess acceptability of the intervention. Three questions requiring likert responses were included in the questionnaires as a result:

1. I feel I benefitted from the consultation.
2. I was able to ask all the questions I wanted to.
3. I would recommend a pharmacist pain medicines consultation to other people.

Data was also collected on whether the community pharmacists completed the consultations when they were requested to do so. The day after the scheduled consultation, the RP telephoned the community pharmacist and gathered unstructured feedback about the experience of providing the consultation.

## Results

128 patients were identified for the study; 47 were confirmed as eligible by their healthcare professional and invited to take part. Twenty-three consented to participate and 19 received the medicines consultations of whom 17 were already receiving specialist palliative care services.^2^ Four patients deteriorated or died before they had consultations. Patients were aged between 33 and 88 (average 64 years old). More detail about recruitment methods is available elsewhere [30].

Four community pharmacists were requested to deliver five consultations, of which all took place. Five patients had a face-to-face MUR from four different community pharmacists (3 independents and 1 multiple) and 14 had two NMS-type consultations from the RP. Five patients were unavailable at the second telephone consultation and required further phone calls. One patient had hearing difficulties and asked for his spouse to be involved in the telephone call to aid communication.

The mean duration of medicines consultations was 31 min for MUR (range 20–60 min) and for the NMS type consultations the mean total time for the two consultations was 18 min (range 9–29 min) per patient.

In total 47 DRPs were identified in 17 patients with a mean of 2.5 per patient (range 0–7, median 2) (see Table 2). Consultations were often multi-faceted (see Exemplar case studies-Box 2) and MURs averaged 1.2 DRPs per patient and the NMS-type averaged 3. Advice was given to 17 patients to resolve 34 DRPs and 13 (for 8 patients) were addressed by referral to other healthcare professionals: 6 to recommend prescribing of additional medication (for pain, constipation or dry mouth), 2 for a concomitant medicines query, 2 to recommend an alternative medicine (for constipation), 1 for an alternative dosage form and 2 to flag up important symptoms to the prescriber.

**Table 2:** Medicine related problems and how they were addressed (n = 19)

Patient	Were they receiving specialist palliative care?	No of MRPs identified	MRP details	PCNE problem	PCNE cause	PCNE inter-vention
Ph8X1	Unknown	2	Advice	P3.3	C5.2	I2.1
			Advice	P3.3	C5.2	I2.1
Ph4X1	Y	2	Pain—paracetamol	P1.2	C7.1	I2.1
			Constipation	P1.3	C1.6	I2.1
Ph8X2	Y	0	–	–	–	–
Ph10X1	N	0	–	–	–	–
Ph9X1	Y	2	Adjuvant dosage	P1.2	C5.2	I2.1
			Constipation^a^	P1.3	C1.6	I1.3
MC5	Y	3	Pain	P1.1	C3.5	I2.1
			Compliance	P1.2	C7.1	I2.1
			Advice	P3.3	C5.2	I2.1
MC6	Y	3	Pain—ibuprofen	P1.2	C7.1	I2.1
			Pain—paracetamol	P1.2	C7.1	I2.1
			Breathlessness	P1.3	C5.2	I2.1
MC7	Y	3	Compliance	P1.2	C7.7	I2.1
			Pain—paracetamol^a^	P1.2	C1.6	I1.4
			Other medication^a^	P1.3	C7.1	I1.4
MC8	Y	2	Drug form^a^	P1.2	C2.1	I1.3
			Side effects	P2.1	C5.2	I2.1
MC9	Y	2	Pain—Paracetamol	P1.2	C7.1	I2.1
			Side effects	P2.1	C5.2	I2.1
MC10	N	2	Pain—paracetamol	P1.2	C7.1	I2.1
			Compliance	P1.2	C7.1	I2.1
MC11	Y	1	Constipation	P1.2	C7.1	I2.1
MC12	Y	5	Pain	P1.2	C7.7	I2.1
			Side effect	P2.1	C5.2	I2.1
			Constipation	P1.2	C7.1	I2.1
			Side effect	P2.1	C5.2	I2.1
MC13	Y	3	Pain^a^	P1.2	C3.1	I1.3
			Laxative^a^	P1.2	C3.1	I1.4
			Advice	P3.3	C5.2	I2.1
MC14	Y	1	Other medication	P1.2	C7.7	I2.1
BRI1	Y	3	Pain	P1.2	C7.1	I2.1
			Constipation^a^	P1.3	C1.6	I1.3
			Medicines sourcing	P3.3	C5.2	I2.1
			Side effect^a^	P2.1	C5.2	I2.1
MC15	Y	2	Pain	P1.2	C7.1	I2.1
			Other medication issues^a^	P3.2	C1.5	(I1.4, I3.5)
MC16	Y	5	Pain—morphine	P1.2	C7.1	I2.1
			Pain—paracetamol	P1.2	C7.1	I2.1
			Constipation	P1.2	C7.1	I2.1
			Side effects^a^	P1.3	C1.6	I1.4
MC17	Y	7	Pain^a^	P1.3	C1.6	I1.3
			Pain^a^	P1.3	C1.6	I1.3
			Advice	P3.3	C5.2	I2.1
			Side effects	P1.2	C5.2	I2.1
			Advice	P3.3	C5.2	I2.1
			Constipation	P1.2	C7.1	I2.1
			Constipation^a^	P1.2	C1.6	I1.4

^a^Indicates MRPs which were referred to another healthcare professional

Full details of the PCNE classification can be found in “Appendix 1” [21].

### Feedback from patients

Eleven patients returned both baseline and post-consultation questionnaires. The answers to the three acceptability questions are shown in Table 3 along with other questions regarding pain and self-reported knowledge.

**Table 3:** Participants’ baseline and follow-up questionnaire responses including acceptability data (n = 11)

	Pre-intervention average pain score (0–10)	Post-intervention average pain score (0–10)	Pre-intervention “Do I know enough about my medicines?”	Post intervention “Do I know enough about my medicines?”	I feel I benefited from the consultation?	I was able to ask all the questions I wanted to?”	I would recommend a pharmacist pain medicines consultation to other people?
Ph8X2	0.5	6.0	Don’t know	Yes	Agree	Agree	Agree
Ph10X1	0.0	0.0	Yes	Not answered	Strongly agree	Strongly agree	Strongly agree
Ph9X1	6.5	7.5	No	No	Neutral	Agree	neutral
MC6	2.0	1.5	Don’t know	Yes	Neutral	Agree	neutral
MC7	3.0	3.5	No	No	Disagree	Not answered	Not answered
MC9	4.0	7.0	Yes	Yes	Agree	Strongly agree	Strongly agree
MC10	4.0	2.5	Yes	Yes	Agree	Agree	Agree
MC11	3.5	1.5	No	No	Neutral	Agree	Agree
MC14	6.0	6.0	No	Yes	Strongly agree	Strongly agree	Strongly agree
MC15	3.0	3.5	No	Yes	Strongly agree	Strongly agree	Strongly agree
MC16	5.0	4.5	No	Yes	Agree	Agree	Agree

Unknown is stated where questionnaires were not returned

Pre-consultations, the mean pain score was 4.1 (range 0–8) and three patients felt they knew enough about their medicines compared with 4.0 and seven at follow-up. No other medicines education support was reported by patients during the intervention period.

### Feedback from community pharmacists

At the follow-up phone call after the consultations three of the four community pharmacists reported having some challenges in carrying out the consultations. Two reported lack of confidence and three had difficulties in retaining knowledge when the consultations were so infrequent.

### Difficulties with recruitment

Several methods of recruitment were used of which one (hospice) produced 18 of the 23 participants. Face to face recruitment methods were found to be more effective than by letter and recruitment was more successful where healthcare professionals were engaged in the study. Full details and evaluation of methods used are reported elsewhere [30].

**Box 2:** Exemplar patient case studies

*Case 1* Patient MC13 who was taking multiple medicines, was discharged from the hospice after several weeks of symptom control. The patient received the NMS style intervention but felt that it would have been more useful before their inpatient stay. At consultation 1 the patient only had a few questions about their medication. At consultation 2 (2 weeks later) their pain had changed, they were struggling with control, using seven top-up doses of strong opioid each day and severely constipated. The patient reported struggling psychologically with others’ perceptions of their pain. Other issues discussed included getting the best use from currently prescribed medicines. A referral was made to the patient’s usual healthcare professional for a suggested increase in slow-release strong opioid and a change in constipation medication. The pharmacist was asked by the healthcare professional to recommend medication for constipation and to investigate its availability. *Case 2* When patient MC5 was contacted for the first NMS-style consultation they were in severe pain and had not been taking their medication as they were in “too much pain” with the pain affecting their ability to think, sleep and function. Paracetamol and tramadol had been prescribed but the patient was not taking paracetamol as they thought their condition was beyond that. The pharmacist explained about taking pain medication on a regular basis and how this could prevent large spikes in pain, and that the effects of paracetamol could make a difference. The patient was concerned about transitioning to strong opioid medication in the future and the associated risk of addiction. This was discussed at length. At the second consultation 9 days later, the patient had started taking more regular pain relief, including a new prescription for morphine sulphate liquid and reported great improvement.	

## Discussion

This study shows that even for patients receiving specialist palliative care, pharmacist-delivered medicines consultations were feasible and acceptable to patients and had the potential to benefit clinical care.

### Feasibility

We found that identification of patients was more difficult than expected so additional methods were developed iteratively. Recruitment and attrition rates were in line with other similar studies [31, 32].

Community pharmacists found it difficult to retain working knowledge regarding cancer and this could potentially be improved if the consultations were carried out more frequently. Creation of referral pathways to community pharmacy were not successful, therefore we also tested telephone provision of medicines consultations by one centralised RP. This was used successfully with a broad age range of patients.

We know from previous research that one in three patients are never referred to specialist palliative care services and we hypothesise that these patients may have greater need for a medicines consultation [4, 33]. Recruitment methods used were less successful in finding those who had not been referred to palliative care. Even though almost all our participants were receiving this; a mean of 2.5 DRPs per patient was found showing a need for extra support even in this group.

### Acceptability to patients

All patients who had an NMS-type service (n = 14) agreed to the second consultation after having the first so we deduce that patients found them acceptable.

The majority of consultations were carried out via telephone. This method was acceptable for all patients who received it and some studies show this may even be preferable for some, especially those who are seriously ill [4, 34]. Telephone-based appointments are already used in many community pharmacies and family doctor practices with high levels of patient satisfaction [35–37].

Retrospective acceptability can be estimated by perceived effectiveness and self-efficacy. Most patients felt they benefitted from the consultations (which was also found elsewhere), were able to ask all the questions they wanted to and would recommend it to others [35]. There was an increase in patients who felt they knew enough about their medicines following the intervention indicating that knowledge was increased. Pain levels in this patient group can change rapidly due to the nature of the illness although average pain levels remained the same [22]. This may be due to a negation in the expected deterioration over time although on such a small sample it is difficult to draw any such conclusions. Patient evaluation is more likely to be obtained following a one-off intervention so this may have affected our response rates [38].

### Acceptability to healthcare professionals

One community pharmacy (n = 10) declined to be involved but all 9 who agreed sent representatives to the voluntary training showing prospective acceptability was generally good. There were a mix of independent and multiple community pharmacies showing a willingness of both groups to take part.

Only one pharmacist (other than the RP) was asked to carry out more than one consultation and although they agreed, this is not enough to signify acceptability at this stage.

### Potential to benefit clinical care

As in other studies the most common DRP identified was pain and several participants were not taking simple painkillers as recommended because they had not been prescribed [11, 17]. The next most identified DRP was constipation, again a finding in other studies [16, 17, 20]. Almost three quarters of DRPs concerned treatment effectiveness. Seventeen MRPs related to patients not understanding how and why to take medications after they had been prescribed. In some cases, medication was ineffective, and the patient required a stronger dose or a change in treatment.

Pharmacists were able to resolve the majority of DRPs with the patient; eight of the 19 patients were referred to nurses or GPs. Many of the referrals would have been prevented if the pharmacist conducting the consultations had been a prescriber with access to medical records. In several previous studies, the pharmacist was either a trained prescriber or was part of a palliative care team that could organise changes in prescribing [17–20, 39]. Acceptance of DRP recommendations by prescribers was unknown and future studies need to track this.

### Limitations of the study

Most patients taking part already had access to palliative care professionals and associated medicines support. If patients had been recruited before referral to palliative care, there may have been an opportunity to educate at an earlier stage.

Patients receiving two consultations were hospice outpatients who had already been referred to palliative care and therefore are more likely to have greater need for symptom control; this may have affected the type and number of DRPs found compared with those who had not been referred. It may be that this group would have more DRPs than those not yet referred to palliative care or it may be that they would have already had more opportunity to get DRPs addressed. This would benefit from further testing across both patient groups.

Acceptability was measured before, during and after the consultations. The numbers of participants, pharmacists and healthcare professionals returning questionnaires was small and this may affect the validity of the results.

## Conclusion

The consultations were feasible to deliver, and patients found them acceptable. Community pharmacists were willing to provide these services although found working knowledge to be problematic due to the infrequent nature of the consultations. Further evaluation of clinical and cost-effectiveness is now needed.

Pharmacist medicines consultations were able to identify a substantial number of DRPs in patients with advanced cancer pain. Problems with inadequate pain relief and associated side effects were most prevalent and the majority of these could be addressed by the pharmacist even in patients already receiving specialist palliative care.

## Appendix 1

See Table 4.

**Table 4:** PCNE classification scheme for drug-related problems V8.02 [21]

Primary domain	Code	Problem
The problems		
1. Treatment effectiveness	P1.1	No effect of drug treatment
	P1.2	Effect of drug treatment not optimal
	P1.3	Untreated symptoms or indication
2. Treatment safety	P2.1	Adverse drug event (possibly) occurring
3. Others	P3.1	Problem with cost-effectiveness of the treatment
	P3.2	Unnecessary drug-treatment
	P3.2	Unclear problem/complaint

Primary domain	Code	Cause
The causes		
1. Drug selection	C1.1	Inappropriate drug according to guidelines/formulary
	C1.2	Inappropriate drug (contra-indicated)
	C1.3	No indication for drug
	C1.4	Inappropriate combination of drugs (inc. herbal)
	C1.5	Inappropriate duplication of therapeutic group/active ingredient
	C1.6	No drug treatment in spite of existing indication
	C1.7	Too many drugs prescribed for this indication
2. Drug form	C2.1	In appropriate drug form (for this patient)
3. Dose selection	C3.1	Drug dose too low
	C3.2	Drug dose too high
	C3.3	Dosage regiment not frequent enough
	C3.4	Dosage regiment too frequent
	C3.5	Dose timing instructions wrong, unclear or missing
4. Treatment duration	C4.1	Duration of treatment too short
	C4.2	Duration of treatment too long
5. Dispensing	C5.1	Prescribed drug not available
	C5.2	Necessary information not provided
	C5.3	Wrong drug. Strength or dosage advised (over the counter)
	C5.4	Wrong drug or strength dispensed
6. Drug use process	C6.1	Inappropriate timing of administration and/or dosing intervals
	C6.2	Drug under-administered
	C6.3	Drug over-administered
	C6.4	Drug not administered at all
	C6.5	Wrong drug administered
	C6.6	Drug administered via wrong route
7. Patient related	C7.1	Patient uses/takes less drug than prescribed or does not take the drug at all
	C7.2	Patient uses/takes more drug than prescribed
	C7.3	Patient abuses drug (unregulated overuse)
	C7.4	Patient uses unnecessary drug
	C7.5	Patient takes food that interacts
	C7.6	Patient stores drug inappropriately
	C7.7	Inappropriate tining or dosing intervals
	C7.8	Patient administers/uses the drug in a wrong way
	C7.9	Patient unable to use drug/form as directed
8. Other	C8.1	No or inappropriate outcome monitoring
	C8.2	Other cause; specify
	C8.3	No obvious cause

Primary domain	Code	Intervention
The planned interventions		
No intervention	I0.1	No intervention
1. At prescriber level	I1.1	Prescriber informed only
	I1.2	Prescriber asked for information
	I1.3	Intervention proposed to prescriber
	I1.4	Intervention discussed with prescriber
2. At patient level	I2.1	Patient (drug) counselling
	I2.2	Written information provided (only)
	I2.3	Patient referred to prescriber
	I2.4	Spoken to family member/caregiver
3. At drug level	I3.1	Drug changed to…
	I3.2	Dosage changed to…
	I3.3	Formulation changed to…
	I3.4	Instructions for use changed to…
	I3.5	Drug stopped
	I3.6	New drug started
4. Other intervention or activity	I4.1	Other intervention (specify)
	I4.2	Side effect reported to authorities

Primary domain	Code	Implementation
Acceptance of the intervention proposals		
1. Intervention accepted (by prescriber or patient)	A1.1	Intervention accepted and fully implemented
	A1.2	Intervention accepted, partially implemented
	A1.3	Intervention accepted but not implemented
	A1.4	Intervention accepted, implementation unknown
2. Intervention not accepted (by prescriber or patient)	A2.1	Intervention not accepted: not feasible
	A2.2	Intervention not accepted: no agreement
	A2.3	Intervention not accepted: other reason
	A2.4	Intervention not accepted: unknown reason
3. Other	A3.1	Intervention proposed, acceptance unknown
	A3.2	Intervention not proposed

## Footnotes

1 Practice pharmacist refers to a role within family doctor or General Practitioner (GP) practices which aids with prescribing, audit, costing and sometimes performing clinical roles. They were involved in this study to allow electronic records to be checked to identify patients and assess eligibility criteria.

2 Specialist Palliative Care Services are received by patients who have been referred and usually involves access to multidisciplinary palliative care healthcare professionals to provide symptom control.
